# Prediction of municipality-level winter wheat yield based on meteorological data using machine learning in Hokkaido, Japan

**DOI:** 10.1371/journal.pone.0258677

**Published:** 2021-10-18

**Authors:** Keach Murakami, Seiji Shimoda, Yasuhiro Kominami, Manabu Nemoto, Satoshi Inoue

**Affiliations:** 1 Hokkaido Agricultural Research Center (HARC), Toyohira, Sapporo, Hokkaido, Japan; 2 National Agriculture and Food Research Organization (NARO), Memuro, Kasai, Hokkaido, Japan; Universiti Teknologi Malaysia, MALAYSIA

## Abstract

This study analyzed meteorological constraints on winter wheat yield in the northern Japanese island, Hokkaido, and developed a machine learning model to predict municipality-level yields from meteorological data. Compared to most wheat producing areas, this island is characterized by wet climate owing to greater annual precipitation and abundant snowmelt water supply in spring. Based on yield statistics collected from 119 municipalities for 14 years (*N* = 1,516) and high-resolution surface meteorological data, correlation analyses showed that precipitation, daily minimum air temperature, and irradiance during the grain-filling period had significant effects on the yield throughout the island while the effect of snow depth in early winter and spring was dependent on sites. Using 10-d mean meteorological data within a certain period between seeding and harvest as predictor variables and one-year-leave-out cross-validation procedure, performance of machine learning models based on neural network (NN), random forest (RF), support vector machine regression (SVR), partial least squares regression (PLS), and cubist regression (CB) were compared to a multiple linear regression model (MLR) and a null model that returns an average yield of the municipality. The root mean square errors of PLS, SVR, and RF were 872, 982, and 1,024 kg ha^−1^ and were smaller than those of MLR (1,068 kg ha^−1^) and null model (1,035 kg ha^−1^). These models outperformed the controls in other metrics including Pearson’s correlation coefficient and Nash-Sutcliffe efficiency. Variable importance analysis on PLS indicated that minimum air temperature and precipitation during the grain-filling period had major roles in the prediction and excluding predictors in this period (i.e. yield forecast with a longer lead-time) decreased forecast performance of the models. These results were consistent with our understanding of meteorological impacts on wheat yield, suggesting usefulness of explainable machine learning in meteorological crop yield prediction under wet climate.

## 1. Introduction

Crop yield prediction models are undoubtedly required for agricultural practices. Statistical regression methods, including multiple linear regression, have been sometimes used for crop yield prediction in earlier studies. Despite its intuitive clarity and ease of practical application in agriculture, the use of this method requires caution because 1) the response of yield to climate variables may be nonlinear and 2) strong correlations among independent (predictor) variables may provide biased interpretations [1, 2]. With increased available data sources and predictor variables, studies have attempted to predict crop yield using machine learning instead of the multiple linear regression method [3]. Several earlier studies applied artificial neural networks for crop yield prediction. Jiang et al. [4] developed an artificial neural network model with remotely sensed variables for wheat yield prediction in Henan province, China. Alvarez [5] used a neural network model based on meteorological and soil data to predict wheat yield in Argentine Pampas with a root mean square error (RMSE) of 450 kg ha^−1^ (mean yield: 2,500 kg ha^−1^). Khashei-Siuki et al. [6] applied artificial neural network and adaptive neuro-fuzzy inference systems to predict dryland wheat yields from meteorological data in Iran, achieving an RMSE value of 151.9 kg ha^−1^ (mean yield: 415.1355 kg ha^−1^). More recently, other machine learning approaches including deep learning have been examined to improve model performance. Kamir et al. [7] compared several base learners including random forest, support vector machine, and k-nearest neighbor algorithm using climate records and satellite image time series to predict Australian wheat yield. In addition, Goméz et al. [8] compared the performance of eight machine learning models to predict wheat yield at a municipal level in Mexico. Wolanin et al. [9] applied a convolutional neural network, ridge regression, and random forest to predict wheat yield in the Indian Wheat Belt. Wang et al. [10] proposed a model consists of long short-term memory and convolution neural networks to predict winter wheat yield at county level in China (RMSE: 721 kg ha^−1^). Cao et al. [11] built and compared yield estimators for winter wheat using random forest and three deep learning models (deep neural network, 1D convolutional neural network, and long short-term memory network) at county and field scales in China. Machine learning techniques have also been utilized to predict yields of other crops [e.g. 12, 13] and to monitor evapotranspiration for water resources engineering [e.g. 14, 15]. Some of these studies have analyzed the importance of predictor variables to improve models’ interpretability and predictive performance [7, 9, 11, 12]. In addition, several studies used a hybrid approach to predict crop yield. Feng et al. [16] simulated plant biomass using a plant growth biophysical model APSIM and combined it with meteorological data and remotely sensed vegetation index as predictors of machine learning models to predict wheat yield in south-east Australia. Shahhosseini et al. [17] also extended the APSIM model to calculate various features related to plant growth and used them and other features as inputs for machine learning models to predict corn yield in the US.

Although earlier studies used various models and succeeded in predicting yields of wheat and other crops, these studies also suggested that base learners and predictors should be properly selected to enhance the performance of models. For example, Gouache et al. [18] developed models for departmental-level wheat yield prediction in France using a stepwise multiple regression method. They selected predictor variables from candidates generated by phenological and water balance models and found that the weights of selected variables varied depending on the department [18]. Because most winter wheat-producing regions in the world are dry (e.g., the Australian Wheatbelt, the Great Plains in the US, and the North China Plain), models proposed in earlier studies may be optimized to wheat yield prediction under dry climate with emphasis on yield loss caused by drought stress in these regions [e.g. 19]. Therefore, these models and deduced meteorological constraints on winter wheat yield may not be valid in wet regions. Because global warming accompanies increases in global mean precipitation and regional precipitation except for semi-arid areas [20], wheat production under wetter regions will rise in the future. To hedge risks caused by climate change and to ensure global food security, analyses on climatic effects on wheat yields and development of yield prediction models are necessary for both dry and wet regions.

This study aimed to analyze meteorological constraints on winter wheat yield and to develop a yield prediction model based on meteorological data, focusing on a Japanese northern island Hokkaido. Hokkaido is a leading wheat producer in Japan and characterized by wet conditions with annual precipitation greater than 1,000 mm in most areas and abundant snowmelt water supply in spring. In this region, precipitation during the wheat growth period has a negative influence on grain yield (see section 3.1). We collected municipality-level yield statistics and meteorological data at a corresponding spatial resolution, analyzed relationships between meteorological conditions and wheat yield, developed machine learning models, and examined their predictive performance.

## 2. Materials and methods

### 2.1 Study domain and meteorological characteristics

**Fig 1:**
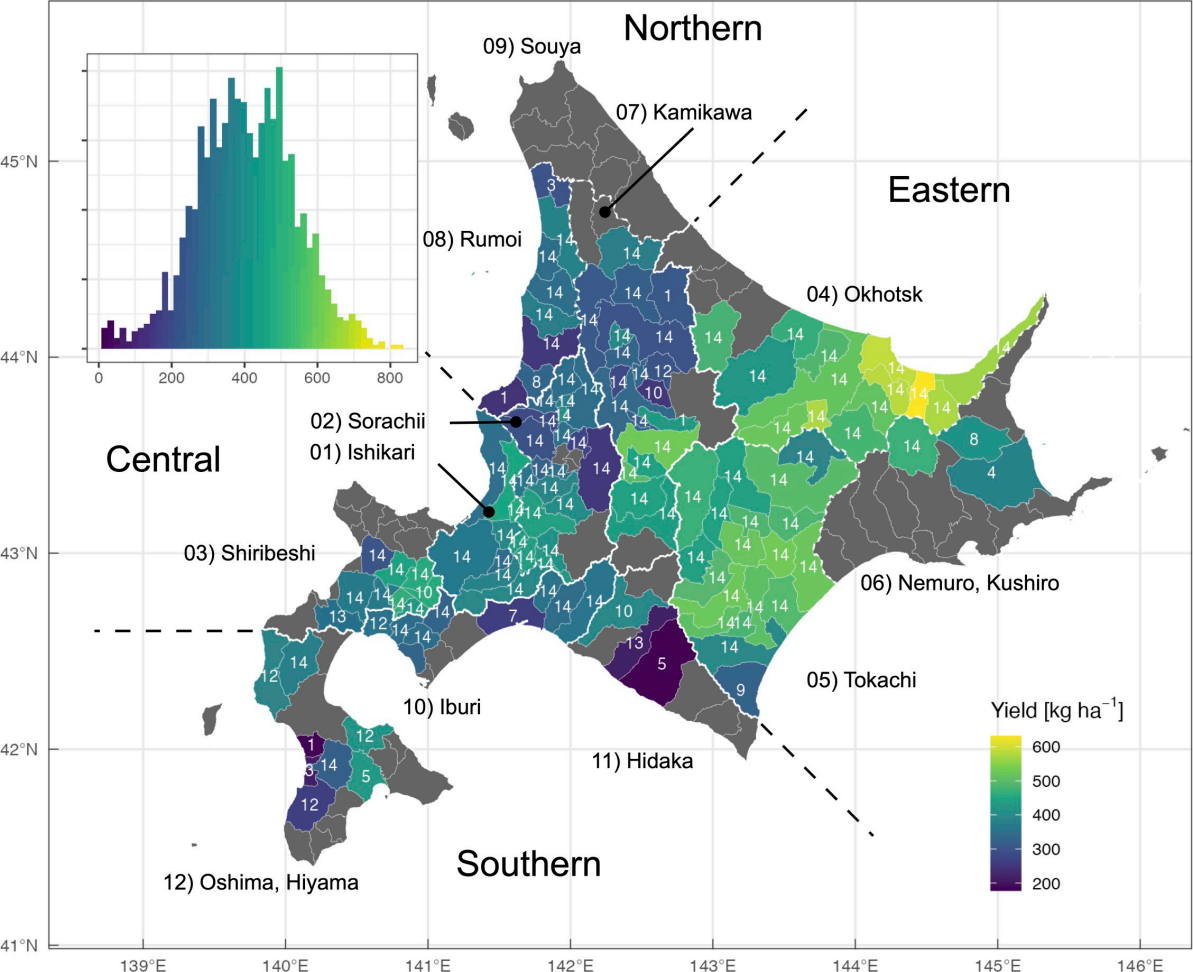
Spatial distribution of municipality-level winter wheat yields used in this study. The number on each municipality (separated by thin lines) indicates the number of years with yield records. Four area groups (separated by thick lines) consist of three or four subprefectures (separated by dashed lines). The inset shows a histogram of the yield (*N* = 1,516). Yield data are published by Hokkaido Regional Agricultural Administration Office (Ministry of Agriculture, Forestry and Fisheries; URL: https://www.maff.go.jp/hokkaido/toukei/kikaku/sokuho/r2kouhyou.html; accessed on 2021 March 31). The boundaries are published by National Land Information Division, National Spatial Planning and Regional Policy Bureau (https://nlftp.mlit.go.jp/ksj/index.html, accessed on 2021-04-06).

The study area was Hokkaido prefecture located in northern Japan, covering approximately 83,000 km^2^ and producing 60% of the Japanese wheat crop. Hokkaido consists of 179 municipalities with a wide range of areas (40–1,400 km^2^). We categorized the municipalities into four groups in this study—eastern, southern, central, and northern groups (Fig 1, Table 1, and S1 Fig in S1 File). The eastern group includes leading wheat production areas (Okhotsk and Tokachi) and is characterized by relatively smaller precipitation and snowfall in Hokkaido. The amounts of annual and growth-period precipitation were still greater than major wheat-producing areas around the world. A large fraction of agricultural areas in the southern and central groups are occupied by paddy fields, and a substantial fraction of wheat fields were formerly used as paddy fields, suggesting poor drainage. The amounts of snowfall are small and great in the south and central, respectively. The northern group is characterized by harsh winter with severe snowfall, and the area of upland fields is small.

**Table 1:** Meteorological statistics of municipalities between 2007 and 2020 in four regional groups.

Region	*N*	*T*_mean_	*T*_min_	*T*_max_	*Rain*	*Irad*	*Snow*
		[°C]	[°C]	[°C]	[mm]	[MJ m^−2^]	[cm]
Central	37	13.1	8.5	18.5	323	2,107	124
		(12.8–13.6)	(8.0–9.0)	(17.9–19.0)	(234–407)	(2,016–2,185)	(91–152)
Eastern	36	11.9	6.8	17.6	346	2,038	74
		(11.4–12.4)	(6.3–7.3)	(16.8–18.3)	(270–407)	(1,977–2,089)	(56–92)
Northern	27	12.7	7.5	18.2	320	2087	111
		(12.3–13.2)	(7.0–8.1)	(17.4–19.0)	(246–384)	(2,020–2,160)	(84–130)
Southern	19	12.9	8.5	17.3	406	2,031	63
		(12.3–13.3)	(7.8–9.2)	(17.6–18.0)	(307–497)	(1,957–2,094)	(38–83)

Number of municipalities (*N*), means and interquartile ranges of mean, minimum, and maximum daily air temperatures (*T*_mean_, *T*_min_, and *T*_max_) and total values of precipitation and irradiance (*Rain* and *Irad*) during the growth period (April to July) and maximum snow depth (*Snow*) are snow.

### 2.2 Dataset

We collected statistical yield records of winter wheat (*Triticum aestivum* L.) at each municipality from 2007 to 2020 in 119 municipalities in Hokkaido (*N* = 1,516; Fig 1) from a Japanese governmental statistical survey by Hokkaido Regional Agricultural Administration Office (Ministry of Agriculture, Forestry and Fisheries; URL: https://www.maff.go.jp/hokkaido/toukei/kikaku/sokuho/r2kouhyou.html; accessed on 2021 March 31). Because some municipalities were merged during this period, we aggregated their records according to the latest district boundary. A dominant winter wheat variety changed from ‘Hokushin’ to ‘Kitahonami’ around 2010, and a new variety ‘Yumechikara’ has covered approximately 10% of the area basis after 2014.

We sourced the Agro-Meteorological Grid Square Data system [21] to collect meteorological data at each municipality. This system provides daily surface-level meteorological data at a grid-spacing of approximately 1 km over Japan. In this study, we used five meteorological elements: minimum and maximum air temperatures (*T*_min_ and *T*_max_), precipitation (*Rain*), global solar irradiance (*Irad*), and snow depth (*Snow*) (Table 1). We used meteorological data from grid cells with 10% or more upland fields to extract *in situ* environmental conditions in upland fields. The land usage information was also provided by the Agro-Meteorological Grid Square Data system.

### 2.3 Machine learning

We used R statistical software (ver. 3.6.2; [22]) for machine learning with a framework implemented by the caret package [23]. This package provides a consistent interface for various models that were developed using other packages in R. To obtain a suitable model, we trained models with the following base learners; multiple linear regression (lm function implemented in base R), model-averaged artificial neural network (avNNet function based on the nnet package; [24]), random forest (rf function based on the randomForest package; [25]), cubist regression (cubist function based on the Cubist package; [26]), support vector machine (svmRadial function based on the kernlab package; [27]), and partial least squares regression (pls function based on the pls package; [28]). The artificial neutral network consists of layered artificial neurons connected to each other with certain weights. This model has been applied in earlier studies for crop yield prediction [e.g. 4–6]. Random forest is an ensemble learning method that creates independent decision trees and returns an average output of these trees [29]. This method requires less computational resources and some studies have adopted random forest in crop yield prediction [e.g. 7, 9]. Cubist regression is a rule-based method that is an extension of Quinlan’s M5 model tree [30]. This is a hybrid approach of decision tree and multiple regression because a tree is grown where the terminal leaves contain linear regression model. Support vector machine constructs a hyperplane in high-dimensional space that robustly separates data into groups by using a subset of training data representative of the groups [31]. In regression analysis with support vector machine, a hyperplane (i.e. no-linear regression model) was determined by minimizing residuals of data outside of an certain margin of errors to avoid overfitting. Partial least squares regression is a statistical method in which linear relationship between projected predictor and predictand matrices in new space [32]. This method was designed to confront the situation that there are many, possibly correlated, predictor variables, and relatively few samples [28].

The predictand was municipality-level yield, and predictors were 10-day mean meteorological data (referred to as early, mid, and late, respectively) and an average yield at the corresponding municipality. We did not use 10-day mean *Snow* data between May and October because they were typically zero. We developed models using yield statistics of municipalities with five or more years (*N* = 1,502).

Models with 10-day meteorological data during various periods between early October (soon after sowing) and late July (just before harvest) were trained and evaluated. The dataset was centerized and scaled before model training to facilitate efficient model tuning. Leave-one-year-out cross-validation was adopted to evaluate the performance of models as follows. We trained models using records of 13 out of 14-year records. Hyperparameters of models were determined by grid search using the train function from the caret package, adopting 10-fold cross-validation using the 13-year dataset (S1 Table in S1 File). Trained models were tested with the remaining 1-year data, and compared to select best combinations of the hyperparameters and input variables. We defined the best model as a model that exhibited the smallest RMSE value among the combinations. The relative importance of the predictor variables was calculated using the varImp function from the caret package. We repeated this procedure for 14 times (i.e., for all years) and obtained 14 RMSE values, root mean squared percentage error (RMSPE), Pearson’s correlation coefficient (*R*), Nash-Sutcliffe efficiency (NSE) [33] and relative importance.

RMSE, RMSPE, and NSE were calculated as follows:

$$\mathrm{RMSE}=\sqrt{\frac{1}{N}\sum^{N}(\hat{Y}-Y{)}^{2}},$$

$$\mathrm{RMSPE}=\sqrt{\frac{100}{N}\sum^{N}{\left(\frac{\hat{Y}-Y}{Y}\right)}^{2}},$$

and

$$\mathrm{NSE}=1-\frac{\sum {\left(\hat{Y}-Y\right)}^{2}}{\sum \left(Y-\bar{Y}\right)},$$

where $\hat{Y}$ and *Y* represent the predicted and observed wheat yield at a certain year and municipality, respectively, $\bar{Y}$ represents an average yield calculated from all records, and *N* represents the number of records. We calculated a relative yield normalized to an average yield of a given municipality as follows:

$$\mathrm{relative}\;\mathrm{yield}=\frac{Y_{i,t}}{\frac{\sum_{t}^{N_{i}}Y_{i,t}}{N_{i}}},$$

where *Y*_i,t_ is an absolute value of wheat yield at municipality *i* and year *t* and *N*_i_ is the number of records at the municipality.

## 3. Results and discussion

### 3.1 Historical and regional variations in winter wheat yield and meteorological constraints

**Fig 2:**
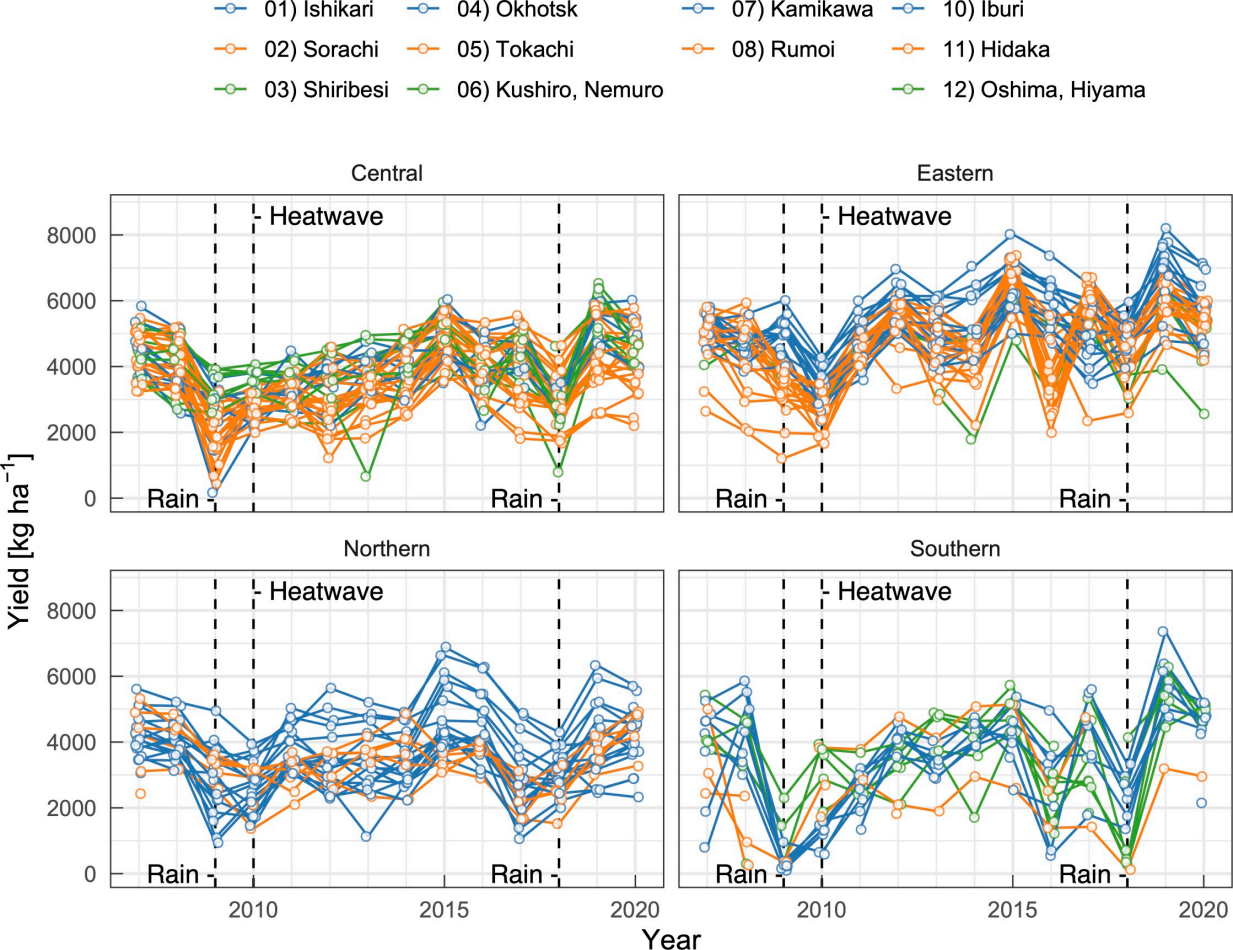
Historical trend of municipality-level winter wheat yield in Hokkaido. Vertical lines represent years with low yields due to a national scale heatwave (2010) and severe rainfall events in summer (2009 and 2018).

Municipality-level winter wheat yields ranged from 100 to 8,200 kg ha^−1^ and the mean value was 4,050 kg ha^−1^ (*N* = 1,516). The yield fluctuated depending on the year and region (Fig 2). For instance, in 2015 and 2019, the yield tended to be high in all regions. On the other hand, in the eastern region, the yield dropped sharply in 2010 due to a national-scale heatwave during summer. In most southern and central regions, the smallest yields were recorded in 2009 or 2018 with heavy summer rain. These data indicate that winter wheat yield should be affected by regional-scale meteorology.

**Fig 3:**
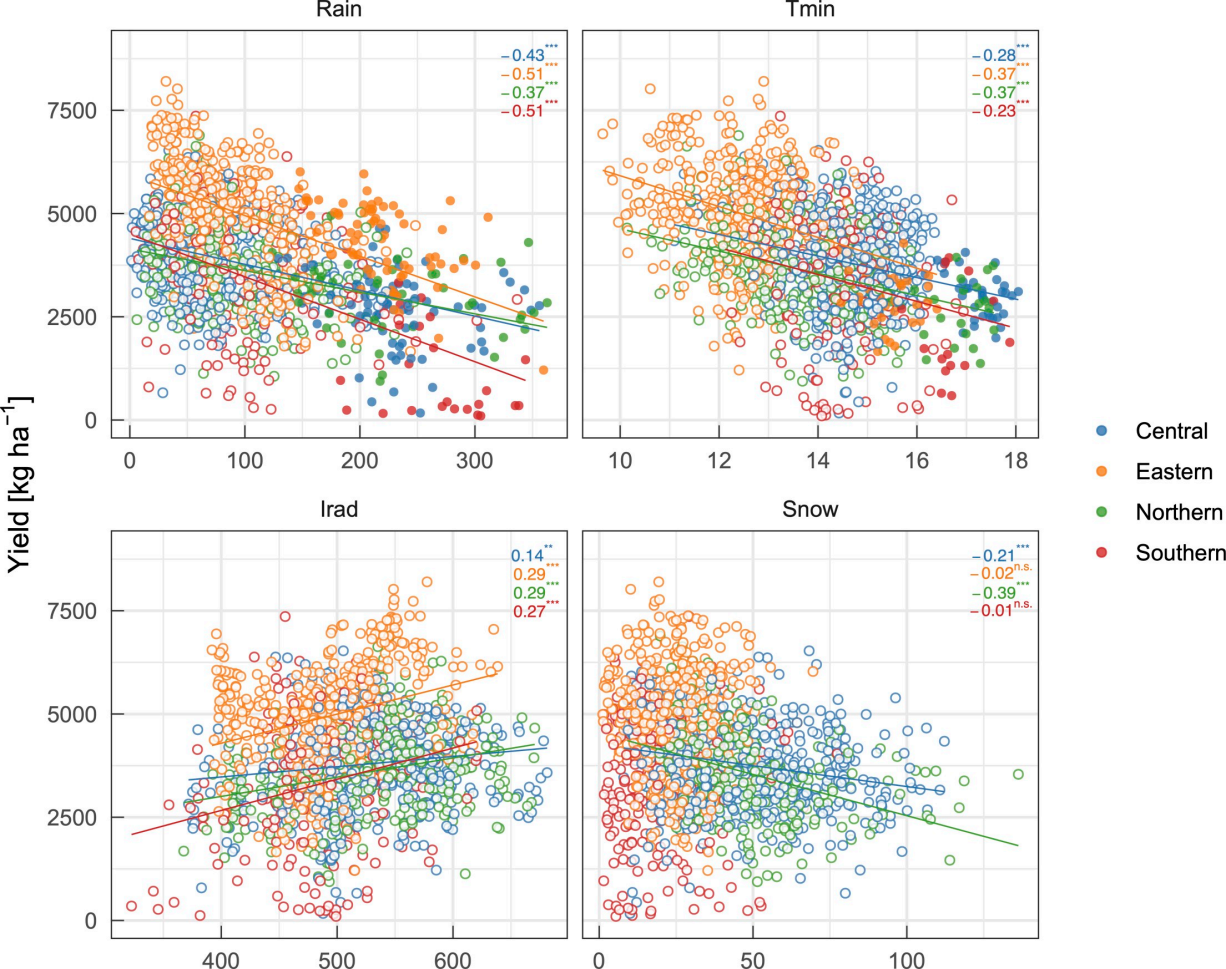
Relationships between the municipality-level yield of winter wheat and total precipitation [*Rain*, mm], mean value of daily minimum air temperature [*T*_min_, °C], and total solar irradiance [*Irad*, MJ m^–2^] during the grain-filling period (mid June to early July), and mean value of snowpack depth [*Snow*, cm] in November and March. Pearson’s correlation coefficients and statistical significance (***: *P* < 0.001, **: *P* < 0.01, *: *P* < 0.05, n.s.: not significant) are shown. Filled symbols in the top-left panel correspond to 2009 and 2018 with severe rainfall events during summer, whereas those in the top-right panel correspond to 2010 with a national-scale heatwave during summer.

Correlation analysis revealed that various environmental factors could be related to the yield (S2 Fig in S1 File). We briefly investigated effects between late June and mid July. This term largely corresponds to the grain filling period before the harvest in most regions in Hokkaido. *Rain* was negatively correlated with the yield in broad regions (Fig 3). During this period, intensive and prolonged rainfall events sometimes induce preharvest sprouting, which degrades grain quality and decreases salable yield [34]. This is contrasting with other wheat-producing regions around the world, where drought often reduces the yield. For example, drought stress during the grain-filling period reduced grain yields in the US [35], Iran [36], and China [37]. Based on data collected at several branches of a local agricultural research institute, Tanifuji [38] performed a correlation analysis and reported that winter wheat yield was not correlated with precipitation. Typically, scientific research institutes are at sites preferable for crop production and their fields are maintained carefully, leading to a climate-robust field condition. This could explain why there was no correlation between precipitation and yield in the report. On the other hand, commercial farmers’ fields may not always be located in suitable sites or be managed well, particularly in low-yield regions. Negative aspects of rainfall before the harvest may be intensified in those fields and resulted in the negative correlation in the present dataset (Fig 3), suggesting techniques for improving soil drainage (e.g., installation of culverts and use of subsoilers) increase wheat yields particularly in low-yield regions. In addition, the present analyses indicate that certain relationships deduced from the ‘elite’ dataset should not be generalized.

As well as precipitation, *T*_min_ was negatively correlated with the yield (Fig 3), as reported in earlier studies [e.g., 38–40]. It has been generally accepted that a higher air temperature shortens the duration of grain filling, reduces the photosynthetic gain, and results in a smaller grain yield [e.g. 41]. A clearer correlation between *T*_min_ and yield than that between *T*_max_ and yield suggests the importance of nighttime temperature on the physiology and growth of wheat plants [42]. Although the trend was unclear as found in the *Rain* and *T*_min_, *Irad* was positively correlated with the yield (Fig 3). This should be attributable to an increase in photosynthetic gain.

While aforementioned environmental elements seemed to affect wheat yield irrespective of regions, *Snow* in early winter and spring (November and March) had contrasting effects on the yield in a site-dependent manner. While more snowfall and thicker snowpack in northern and central regions could decrease wheat yield, snowfall in the eastern and southern regions does not seem to affect wheat yield (Fig 3). In their mesoscale meteorological analysis, Shimoda et al. [40] also reported similar site-dependent relationships between environmental factors and winter wheat yield. They focused on the Okhotsk and Tokachi regions, which were the leading producing areas categorized as the eastern group in the present study (Fig 1) and found that high temperatures from mid June to mid July decreased wheat yield particularly when sunshine duration was shorter than 4.5 h and that contrasting patterns of summer sunshine duration in these regions differentiated yearly yield fluctuations [40]. Such site-dependent relationships between environments and crop yield frequently necessitate the development of empirical models tailored to specific regions. In the present study, we adopted machine learning techniques to develop a versatile model for whole regions of the Hokkaido island.

### 3.2 Prediction of municipality-level wheat yield using machine learning techniques

**Fig 4:**
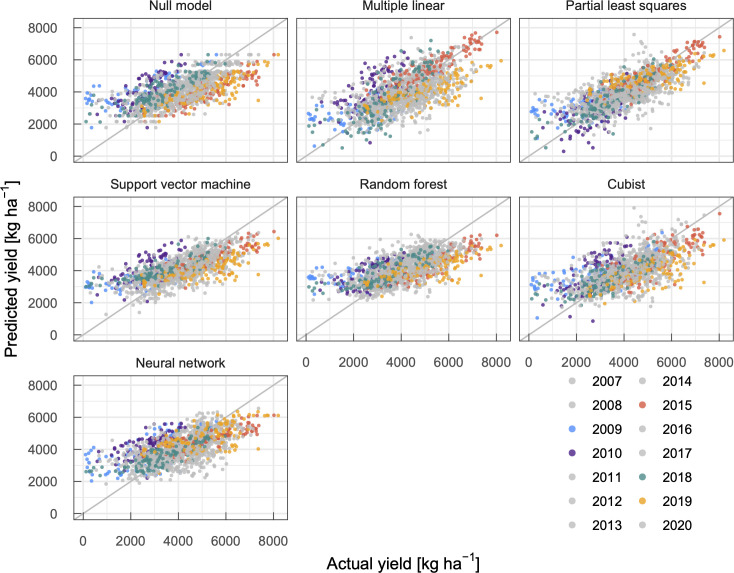
Relationship between actual municipality-level winter wheat yields and predicted ones using machine learning models (*N* = 1,502). Years with high yields (2015 and 2019) and low yields (2009, 2010, and 2018) are highlighted. Evaluation metrics are summarized in Table 2.

**Fig 5:**
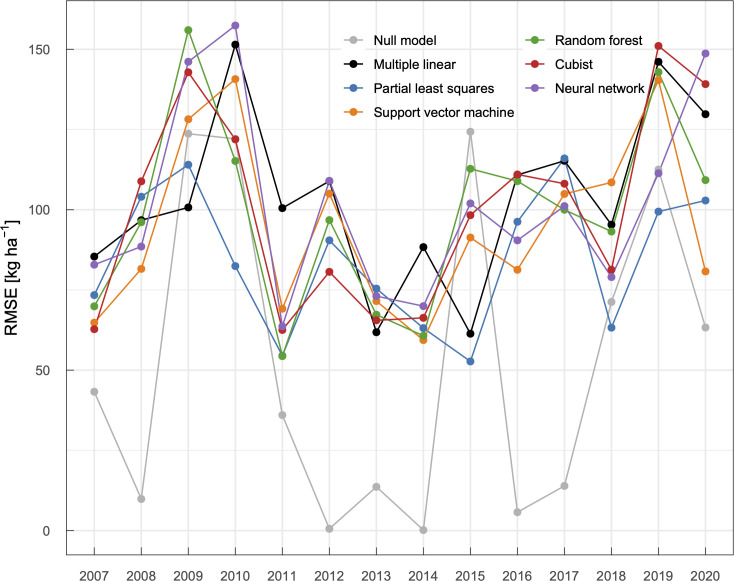
Yearly RMSE values of models for winter wheat yield prediction calculated via leave-one-year-out cross-validation.

We first determined the input period that minimizes the RMSE in predicted yields by varying the start and end of the period (S3 Fig in S1 File). Although there were slight differences among models, models that used meteorological features during the growth period, from April to July, exhibited smaller RMSE values. Because a longer input period (e.g., early October to late July) did not reduce RMSE values (S3 Fig in S1 File), meteorological elements during winter may have smaller effects on year-to-year yield fluctuations compared to those during the growth period. We then compared the prediction performance of models to select the best models, i.e., models that exhibited the smallest RMSE values with respective base learners (Figs 4 and 5, Table 2). Among the models with different base learners, models based on partial least squares and support vector machine showed smaller RMSE values and higher *R* and NSE values, followed by random forest. These models outperformed the null model and multiple linear regression model. While the cubist and neural network models outperformed the multiple linear regression model, their predictive skills were inferior to the null model. Judging from these metrics, the present models were less accurate compared to some latest studies. For example, Wolanin et al. [9] developed machine learning models for wheat yields in the Indian Wheat Belt with a NSE of 0.757–0.868. However, the NSE score for their null model was as high as 0.812, suggesting a smaller year-to-year yield fluctuation in the study domain. It is not always useful to compare metrics of models developed to predict different regions.

**Table 2:** Predictive performance of selected models and their input periods of meteorological data.

Model	RMSE	R	NSE	RMSPE	Input period
	[kg ha^–1^]			[%]	
Null model	1,035	0.64	0.412	16.2	-
	(2–1,243)	(0.354–0.769)	(0.035–0.710)	(1.8–53.8)	
Multiple Linear	1,068	0.65	0.373	11.9	Late March–Early July
	(614–1,515)	(0.309–0.811)	(-0.204–0.877)	(1.6–36.6)	
Partial Least Squares	872	0.76	0.583	12.6	Mid April–Early July
	(527–1,160)	(0.309–0.824)	(-0.034–0.909)	(1.3–41.7)	
Support Vector Machine	982	0.70	0.470	15.0	Late March–Late July
	(594–1,407)	(0.277–0.760)	(0.062–0.728)	(1.6–45.5)	
Random Forest	1,024	0.65	0.424	16.7	Mid April–Mid July
	(544–1,560)	(0.315–0.776)	(0.118–0.696)	(1.8–53.9)	
Cubist	1,041	0.64	0.405	14.7	Early March–Early July
	(625––1,510)	(0.296–0.680)	(-0.385–0.685)	(1.9–47.8)	
Neural Network	1,055	0.63	0.389	15.7	Mid April–Early July
	(636–1574)	(0.332–0.686)	(-0.581–0.696)	(1.8–51.2)	

Root mean square errors (RMSE), Pearson’s correlation coefficient (R), Nash- Sutcliffe efficiency (NSE), and root mean square percent errors (RMSPE) are shown (*N* = 1,502). Values in the parentheses represent ranges of metrics calculated via leave-one-year-out cross-validation.

Although several studies have used linear regression or correlation analysis to discuss environmental effects of meteorological factors on crop yields, several studies have highlighted potential problems [1, 2]. A simple approach with multiple regression should be limited by multicollinearity because of correlations among explanatory variables. For example, there was a strong negative correlation between *Irad* and *Rain* during the grain-filling period (S4 Fig in S1 File). In addition, most biological responses to environments are expressed with nonlinear functions. Other approaches are necessary to address these issues, and the present results exemplified the usefulness of machine learning techniques. Note that these machine learning models can now be examined easily thanks to open-source products and developers’ continuous effort [e.g. 22, 26].

The partial least squares model predicted wheat yields reasonably well, even in low- and high-yield years. For example, this model provided better estimates of high yields in 2015 and 2019 and low yields because of an extraordinarily hot summer (2010) and heavy rainfalls (2009 and 2018) (Fig 5). Although linear correlations between the predicted and actual yields were observed in models based on support vector machine and random forest, the slope was smaller than one (Fig 4). These models tended to return milder estimates and are similar to the null model. A similar trend has been repeatedly reported in earlier studies [e.g. 43]. Continuous data accumulation may improve prediction performance of models since the number of extreme events were small in the training dataset. While the cubist model also showed sensitivity in low- and high-yield years, predictions in years with near-average yields were less reliable. The neural network model exhibited relatively lower performance in terms of sensitivity and stability. As mentioned in the Introduction, a number of studies have applied machine learning methods to predict crop yields and implied that a suitable model should be developed based on purposes, crops, and applied regions. The present results again emphasized the need for an appropriate model selection to improve the predictive performance. Because weather and climate extremes are projected to become more frequent and intensified [44], year-to-year crop yields will fluctuate more drastically. Considering the demand for predicting extreme yields, the partial least squares model may be an optimal solution in the present study.

**Fig 6:**
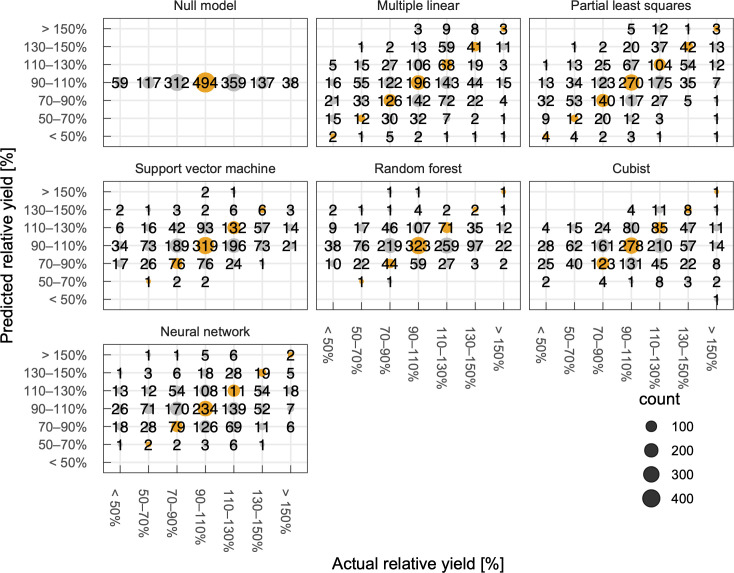
Relationship between predicted and actual relative yields categorized with a bin size of 20%. Circles in orange and gray indicate correct and incorrect classification, respectively. Numbers on and sizes of the circle indicate the number of records in each category.

**Fig 7:**
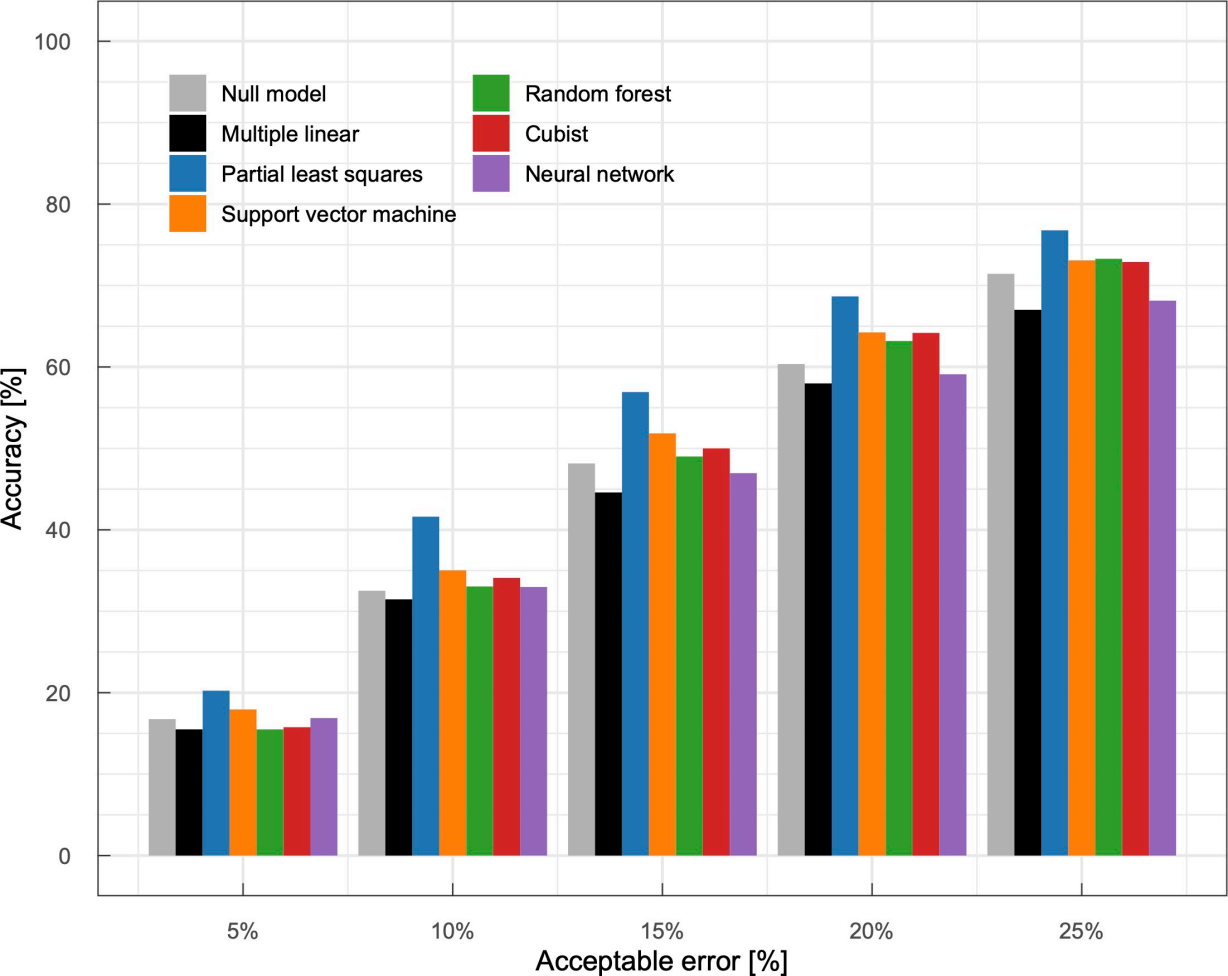
Accuracy of predicted municipality-level winter wheat yields as a function of an acceptable error. Each predicted value was regarded as a correct prediction if | (predicted yield) / (actual yield) − 1| was smaller than the acceptable error.

From a perspective of end users of yield prediction models such as local farmers and municipality administrators, an accuracy of predicted relative yields is of interest because their main concern is to know yields are greater, smaller, or about the same compared to the normal year. To assess the models from a practical perspective, we categorized the predicted and actual yields normalized to the average yield with a bin size of 20% to generate a multi-class confusion matrix (Fig 6). Among the machine learning models, the predicted categories by the partial least squares model were usually the same to or neighboring to the actual categories. The machine learning models tended to overpredict yields in low-yield years and *vice versa* probably because of the smaller number of such conditions in the present dataset. Therefore, the predicted yields were less reliable when actual yields were apart from their average values. To further improve interpretability, we analyzed a relationship between model accuracy and an acceptable error (Fig 7). When we only accept an error of 5% in the predicted yield, the accuracy in all models was as few as 20%. On the other hand, when we accept an error of 25%, the accuracy in the partial least squares model was higher than 75%. Because crop yields at a given site are usually around an average yield at the site, it is natural that the null model exhibits a reasonably high accuracy. Nevertheless, some models—partial least squares, support vector machine, random forest, and cubist—achieved higher accuracy than the null model in most cases of acceptable error, indicating the potential usefulness of these models. Note that the machine learning models usually outperformed the multiple linear regression model.

**Fig 8:**
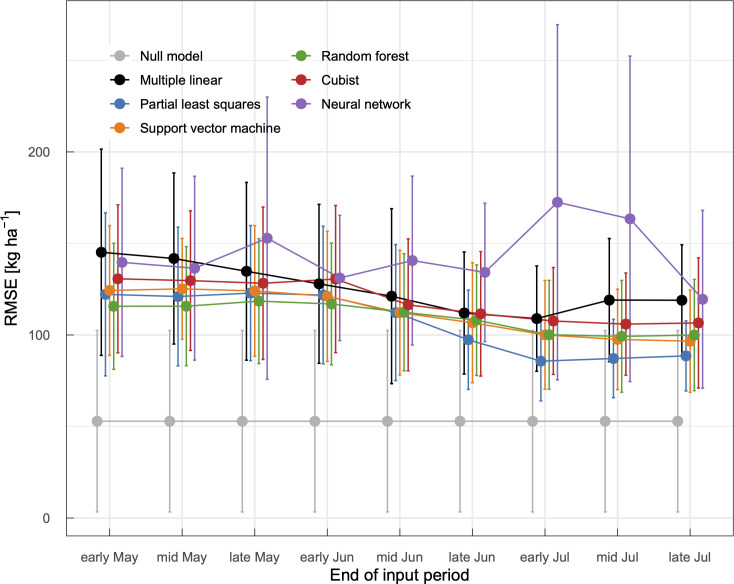
Performance of machine learning models for winter wheat yield forecast. Models use meteorological data from early April to different terms as shown on the x-axis. Mean values and standard deviations calculated via one-year-out cross-validation are shown (*N* = 14).

We also assessed the performance of models in terms of yield forecast (Fig 8). Forecasting crop yields can help stakeholders make economic decisions in advance. To develop models that forecast crop yield before harvest, we trained models by masking meteorological data for a certain period before harvest. An extension in the input period decreased RMSE values in several models including models based on partial least squares, support vector machine, random forest, and cubist. In these models, the decreased forecast RMSE by the extension was marked until early July; it was less marked after mid July. Because photosynthetic biomass accumulation of wheat plants should cease around early July in the present study domain, the variables involved in this process after mid July should have little effect on grain yield. Feng et al. [16] also reported that incorporating predictor variables after flowering did not result in substantial increases in metrics. Considering relationships between growth-period meteorology and wheat yield reported in earlier studies [e.g. 39–40] and shown in the previous section, it is reasonable that incorporating meteorological features around June and July improved model performance. The RMSE values of the multiple linear regression model increased by the extension due to the multicollinearity problem. This model did not show a smaller RMSE value even when it was trained using full-span data (from early April to late July). Similar to the multiple linear regression model, RMSE values of the model based on neural network fluctuated. This might originate from a simple architecture of the present neural network model (a single hidden layer with several nodes), implying that a complicated network is required for an accurate forecast. Because the other base learners such as partial least squares demonstrated validity without meticulous tuning, we should use them for crop yield prediction and forecast.

**Fig 9:**
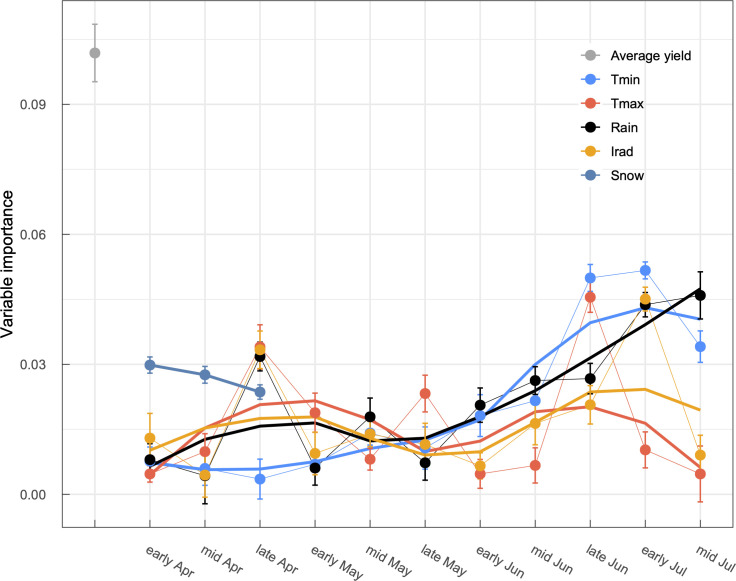
Variable importance of a partial least squares model for winter wheat yield prediction. Mean values and standard deviations calculated via one-year-out cross-validation are shown (*N* = 14). Loess smoothing curves show trends in the variable importance.

We then calculated the variable importance of predictors focusing on the partial least squares model, which exhibited the best performance. Variable importance analysis implied that *T*_min_ during the grain-filling period was the second most important environmental variable next to the municipality-level average yield (Fig 9). A small peak in the importance of *Irad* was found at the same period to *T*_min_. These elements may be involved in the duration of the grain-filling period and photosynthetic gain. A slightly smaller and delayed peak compared to that of *T*_min_ was observed in the importance of *Rain*, suggesting a strong impact of preharvest sprouting on wheat yield [34]. These results are consistent with our current understanding of meteorological effects on wheat yield as discussed in the previous section. The present study confirms that machine learning techniques are useful for prediction of winter wheat yields in wet regions. Considering the remarkable weights of precipitation in the present model and its negative effect on the yield (Fig 3), the present model should not be valid for yield prediction in dry regions. For example, Cao et al. [11] analyzed variable importance for prediction of winter wheat yields in China and found that air temperatures and precipitation during winter had larger effects than those during the growth period because of their close relationships to cold and drought stress on wheat plants. It has been projected that rainfall patterns will change and that annual precipitation will increase in several wheat producing regions including eastern Australia, northern China, and northern Europe [20]. The present analyses and developed models may be a helpful reference to investigate wheat production in these regions under the future climate.

### 3.3 Concluding remark

In this study, we analyzed meteorological constraints of winter wheat yield in Hokkaido, where meteorological conditions during the growth period were wetter than those in other wheat-producing areas worldwide. Our municipality-level analyses revealed an overlooked negative effect of precipitation during the grain-filling period in this region. We developed a machine learning model based on the partial least squares method that predicts and forecasts wheat yield reasonably well. The present comparison among the base learners and input periods emphasizes that appropriate selection of these factors is necessary for developing a machine learning model for crop yield prediction. The variable importance of model predictors was consistent with our understanding of relationships between environmental factors and wheat yield. This finding indicates the usefulness of explainable machine learning in meteorological crop yield prediction. Performance of the present machine learning models should be assessed in combination with weather forecast. Latest weather forecast systems offer numerical forecast more than 10 d at a high spatial resolution [e.g. 45]. In addition, use of deep learning techniques and multisource data such as satellite derived vegetation indices [e.g. 4, 7–11] and soil water management indices [e.g. 14, 15] should be examined to enhance model performance in future works.

## Supporting information

S1 File Supplemental figures. (PDF)

## References

[1] Katz RW. Assessing the impact of climatic change on food production. Climate Change. 1977;1: 85–96.

[2] White JW. Comments on a report of regression-based evidence for impact of recent climate change on winter wheat yields. Agriculture, Ecosystems & Environment. 2009;129: 547–548.

[3] Dokic K, Blaskovic L, Mandusic D. From machine learning to deep learning in agriculture–the quantitative review of trends. IOP Conference Series: Earth and Environmental Science. 2020;614: 012138. doi: 10.1088/1755-1315/614/1/012138

[4] Jiang D, Yang X, Clinton N, Wang N. An artificial neural network model for estimating crop yields using remotely sensed information. International Journal of Remote Sensing. 2004;25: 1723–1732.

[5] Alvarez R. Predicting average regional yield and production of wheat in the Argentine Pampas by an artificial neural network approach. European Journal of Agronomy. 2009;30: 70–77.

[6] Khashei-Siuki A, Kouchkzadeh M, Ghahraman B. Predicting dryland wheat yield from meteorological data using expert system, Khorasan Province, Iran. Journal of Agricultural Science and Technology. 2011;13: 627–640.

[7] Kamir E, Waldner F, Hochman Z. Estimating wheat yields in Australia using climate records, satellite image time series and machine learning methods. ISPRS Journal of Photogrammetry and Remote Sensing. 2020;160: 124–135.

[8] Gómez D, Salvador P, Sanz J, Casanova JL. Modelling wheat yield with antecedent information, satellite and climate data using machine learning methods in Mexico. Agricultural and Forest Meteorology. 2021;300: 108317.

[9] Wolanin A, Mateo-García G, Camps-Valls G, Gómez-Chova L, Meroni M, Duveiller G, et al. Estimating and understanding crop yields with explainable deep learning in the Indian Wheat Belt. Environmental Research Letters. 2020;15: 024019.

[10] Wang X, Huang J, Feng Q, Yin D, Winter wheat yield prediction at county level and uncertainty analysis in main wheat-producing regions of China with deep learning approaches. Remote Sensing. 2020;12: 1744.

[11] Cao J, Zhang Z, Lul Y, Zhang L, Zhang J, Li Z, Wheat yield predictions at a county and field scale with deep learning, machine learning, and google earth engine. 2021 European Journal of Agronomy. 2021;123: 126204.

[12] Khaki S, Wang L, Archontoulis SV, A CNN-RNN framework for crop yield prediction. Frontiers in Plant Science 2020;10: 1750 doi: 10.3389/fpls.2019.01750 32038699 PMC6993602

[13] Jiang Z, Liu C, Ganapathysubramanian B, Hayes DJ, Sarkar S, Predicting county-scale maize yields with publicly available data. Scientific Reports. 2020;10: 14957. doi: 10.1038/s41598-020-71898-8 32917920 PMC7486922

[14] Elbeltagi A, Deng J, Wang K, Malik A, Maroufpoor S, Modeling long-term dynamics of crop evapotranspiration using deep learning in a semi-arid environment. Agricultural Water Management. 2020;241: 106334.

[15] Malik A, Kumar A, Rai P, Kuriqi A, Prediction of multi-scalar standardized precipitation index by using artificial intelligence and regression models. Climate. 2021;9: 28.

[16] Feng P, Wang B, Li Liu D, Waters C, Xiao D, Shi L, et al. Dynamic wheat yield forecasts are improved by a hybrid approach using a biophysical model and machine learning technique. Agricultural and Forest Meteorology. 2020;285: 107922.

[17] Shahhosseini M, Hu G, Huber I, Archontoulis SV. Coupling machine learning and crop modeling improves crop yield prediction in the US Corn Belt. Scientific Reports. 2021;11: 1–15. doi: 10.1038/s41598-020-79139-8 33452349 PMC7810832

[18] Gouache D, Bouchon A-S, Jouanneau E, Le Bris X. Agrometeorological analysis and prediction of wheat yield at the departmental level in France. Agricultural and Forest Meteorology. 2015;209: 1–10.

[19] Lobell DB, Hammer GL, Chenu K, Zheng B, McLean G, Chapman SC. The shifting influence of drought and heat stress for crops in northeast Australia. Global Change Biology. 2015;21: 4115–4127. doi: 10.1111/gcb.13022 26152643

[20] Hanasaki N, Fujimori S, Yamamoto T, Yoshikawa S, Masaki Y, Hijioka Y, et al. Hydrology and Earth System Sciences. 2013;17: 2393–2413.

[21] Ohno H, Sasaki K, Ohara G, Nakazono K. Development of grid square air temperature and precipitation data compiled from observed, forecasted, and climatic normal data. Current Biology. 2016;16: 71–79.

[22] R Core Team. R: A language and environment for statistical computing. Vienna, Austria: R Foundation for Statistical Computing; 2019. Available: https://www.R-project.org/

[23] Kuhn M. caret: Classification and regression training. 2020. Available: https://github.com/topepo/caret/

[24] Venables WN, Ripley BD. Modern applied statistics with S. Fourth. New York: Springer; 2002. Available: http://www.stats.ox.ac.uk/pub/MASS4

[25] Liaw A, Wiener M. Classification and regression by randomForest. R News. 2002;2: 18–22. Available: https://CRAN.R-project.org/doc/Rnews/

[26] Kuhn M, Quinlan R. Cubist: Rule- and instance-based regression modeling. 2020. Available: https://CRAN.R-project.org/package=Cubist

[27] Karatzoglou A, Smola A, Hornik K, Zeileis A. Kernlab–an S4 package for kernel methods in R. Journal of Statistical Software. 2004;11: 1–20. Available: http://www.jstatsoft.org/v11/i09/

[28] Mevik B-H, Wehrens R, Liland KH. Pls: Partial least squares and principal component regression. 2019. Available: https://CRAN.R-project.org/package=pls

[29] Ho TK, Random decision forests, In: Proceedings of 3rd International Conference on Document Analysis and Recognition; 1995. pp. 278–282.

[30] Quilan JR, Learning with continuous classes. In: Adams A, Sterling L, editors. Proceedings of the 5th Australian Joint Conference on Artificial Intelligence; 1992. pp. 343–348.

[31] Cortes C, Vapnik V, Support-vector networks. Machine Learning. 1995;20: 273–297.

[32] Wold S, Sjöströma M, Erikssonb L, PLS-regression: a basic tool of chemometrics. Chemometrics and Intelligent Laboratory Systems. 58;28: 109–130.

[33] Nash JE, Sutcliffe JV, River flow forecasting through conceptual models part I—A discussion of principles. Journal of Hydrology. 1970;10: 282–290.

[34] Mares D. Pre-harvest sprouting in wheat. I. Influence of cultivar, rainfall and temperature during grain ripening. Australian Journal of Agricultural Research. 1993;44: 1259–1272.

[35] Xue Q, Zhu Z, Musick JT, Stewart B, Dusek DA. Physiological mechanisms contributing to the increased water-use efficiency in winter wheat under deficit irrigation. Journal of Plant Physiology. 2006;163: 154–164. doi: 10.1016/j.jplph.2005.04.026 16399006

[36] Saeidi M, Abdoli M. Effect of drought stress during grain filling on yield and its components, gas exchange variables, and some physiological traits of wheat cultivars. Journal of Agricultural Science and Technology. 2015;17: 885–898.

[37] Xu Z, Yu Z, Wang D, Zhang Y. Nitrogen accumulation and translocation for winter wheat under different irrigation regimes. Journal of Agronomy and Crop Science. 2005;191: 439–449.

[38] Tanifuji K. Winter wheat. In: Shiga H, Nakatsuji T, editors. Miscellaneous publication of Hokkaido research organization agricultural experiment stations. Hokunoukai; 2011. pp. 23–31.

[39] Nishio Z, Ito M, Tabiki T, Nagasawa K, Yamauchi H, Hirota T. Influence of higher growing-season temperatures on yield components of winter wheat (*Triticum aestivum* L.). Crop Science. 2013;53: 621–628.

[40] Shimoda S, Hamasaki T, Hirota T, Kanno H, Nishio Z. Sensitivity of wheat yield to temperature changes with regional sunlight characteristics in eastern Hokkaido. International Journal of Climatology. 2015;35: 4176–4185.

[41] Asseng S, Ewert F, Martre P, Rötter RP, Lobell DB, Cammarano D, et al. Rising temperatures reduce global wheat production. Nature Climate Change. 2015;5: 143–147.

[42] Sadok W, Jagadish SK. The hidden costs of nighttime warming on yields. Trends in Plant Science. 2020;25: 644–651. doi: 10.1016/j.tplants.2020.02.003 32526169

[43] Jeong JH, Resop JP, Mueller ND, Fleisher DH, Yun K, Butler EE, et al. Random forests for global and regional crop yield predictions. PLoS ONE. 2016;11: e0156571. doi: 10.1371/journal.pone.0156571 27257967 PMC4892571

[44] IPCC. Managing the Risks of Extreme Events and Disasters to Advance Climate Change Adaptation. A Special Report of Working Groups I and II of the Intergovernmental Panel on Climate Change. In: Field CB, Barros V, Stocker TF, Qin D, Dokken DJ, Ebi KL, Mastrandrea MD, Mach KJ, Plattner GK, Allen SK, Tignor M, Midgley PM. editors, Cambridge University Press; 2012.

[45] Japan Meteorological Agency, Numerical weather prediction models. In: Outline of the operational numerical weather prediction at the Japan Meteorological Agency. 2019. pp. 47–138.

