# Supplementary material for: Prediction of municipality-level winter wheat yield based on meteorological data using machine learning in Hokkaido, Japan
Source: PLoS One. 2021 Oct 18;16(10):e0258677. doi: 10.1371/journal.pone.0258677 (PMC8523044; doi:10.1371/journal.pone.0258677)
Supplement: S1 File — (PDF) [file pone.0258677.s001.pdf]

 doi: 10.1371/journal.pone.0258677

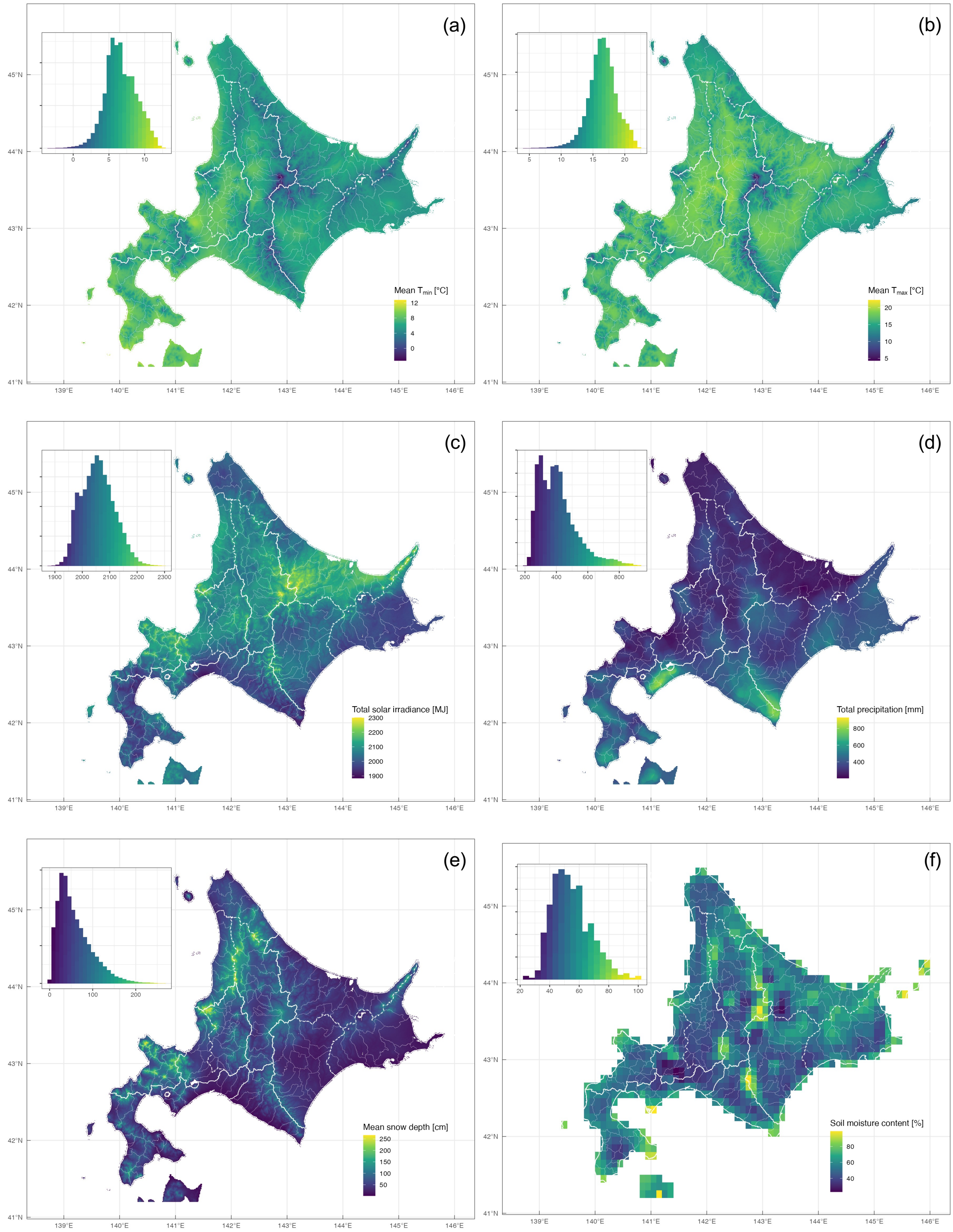

Fig. S1: Spatial distribution of climatological normal values (1981–2010) in (a) daily minimum

air temperature, (b) daily maximum air temperature, (c) global solar irradiance, (d) precipitation, and (e) snow depth and (f) soil moisture content at a grid-spacing of 10 km monitored by satellite remote sensing (band-X of AMSR/GCOM-W1, 2013–2020). Mean or total values during wheat growth period (Apr–July) are shown for (a), (b), (c), (d), and (f) and mean values during winter (Nov–Mar) are shown for (e). The insets show histograms of the variables.

References:

- Vrije Universiteit Amsterdam (Richard de Jeu) and NASA GSFC (Manfred Owe) (2014), AMSR2/GCOM-W1 surface soil moisture (LPRM) L3 1 day 10 km x 10 km descending V001, Greenbelt, MD, USA, Goddard Earth Sciences Data and Information Services Center (GES DISC), Accessed on 2021-02-19 doi:10.5067/SITUTTDUKYZE

- Owe M, de Jeu R, Holmes T. Multisensor historical climatology of satellite-derived global land surface moisture, Journal of Geophysical Research 2008; 113(F01002):1–17. doi:10.1029/2007JF000769.

 doi: 10.1371/journal.pone.0258677

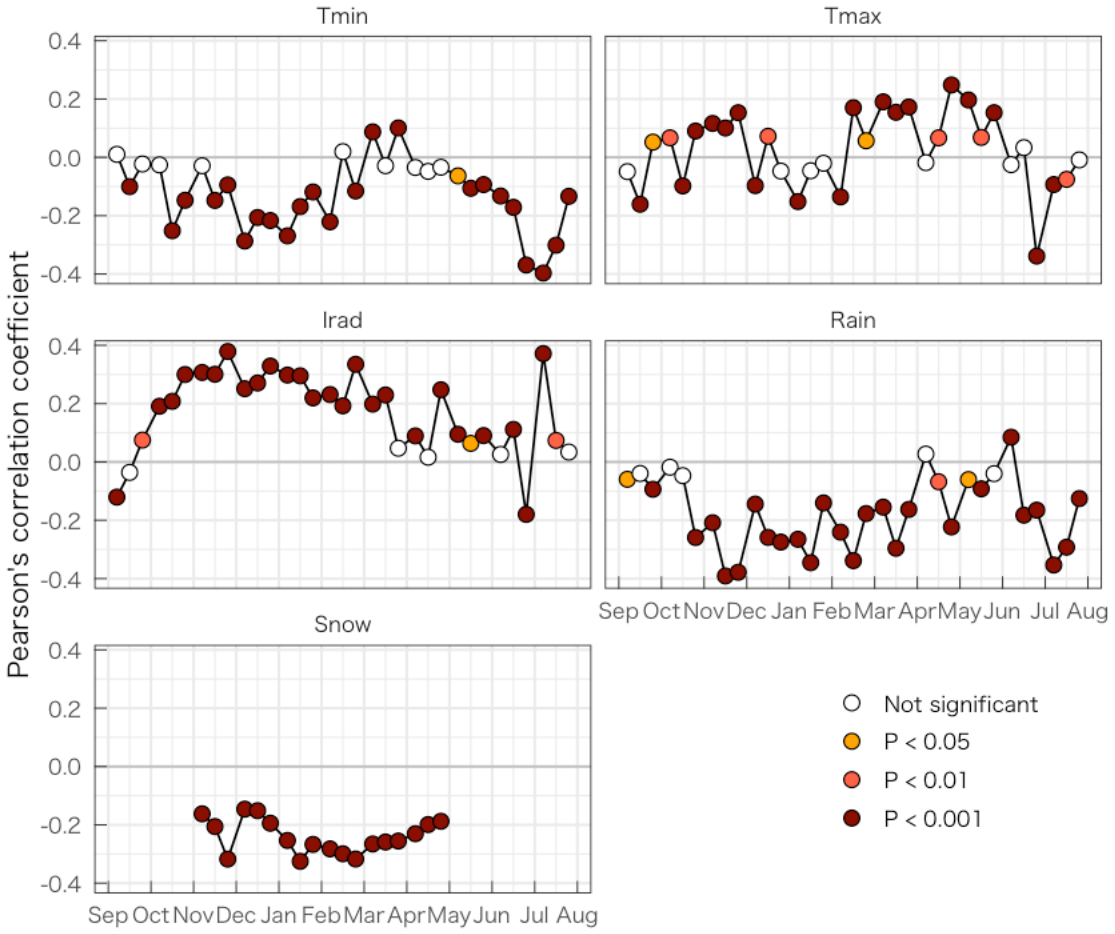

Figure S2: Seasonal changes in correlation between 10-day-mean environments and winter wheat yield. The Pearson's correlation coefficients are shown. Color of the symbols represents P-values.

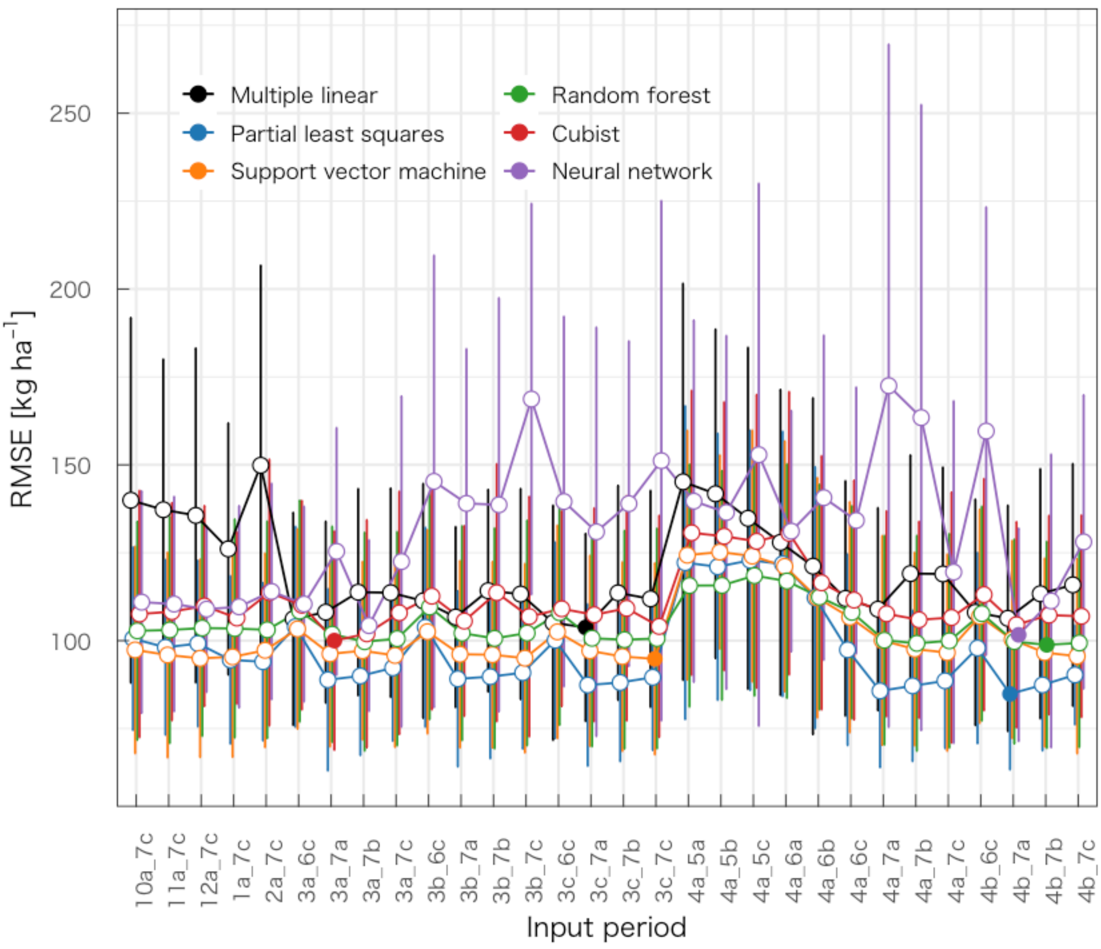

Figure S3: Prediction RMSE values of models which take meteorological variables during different input periods. '4a_6b' indicates that models take the variables between early April and mid June. Filled symbols correspond to models that exhibited the smallest RMSE values. Mean values and standard deviations calculated via one-year-out cross-validation are shown ($N = 14$).

 doi: 10.1371/journal.pone.0258677

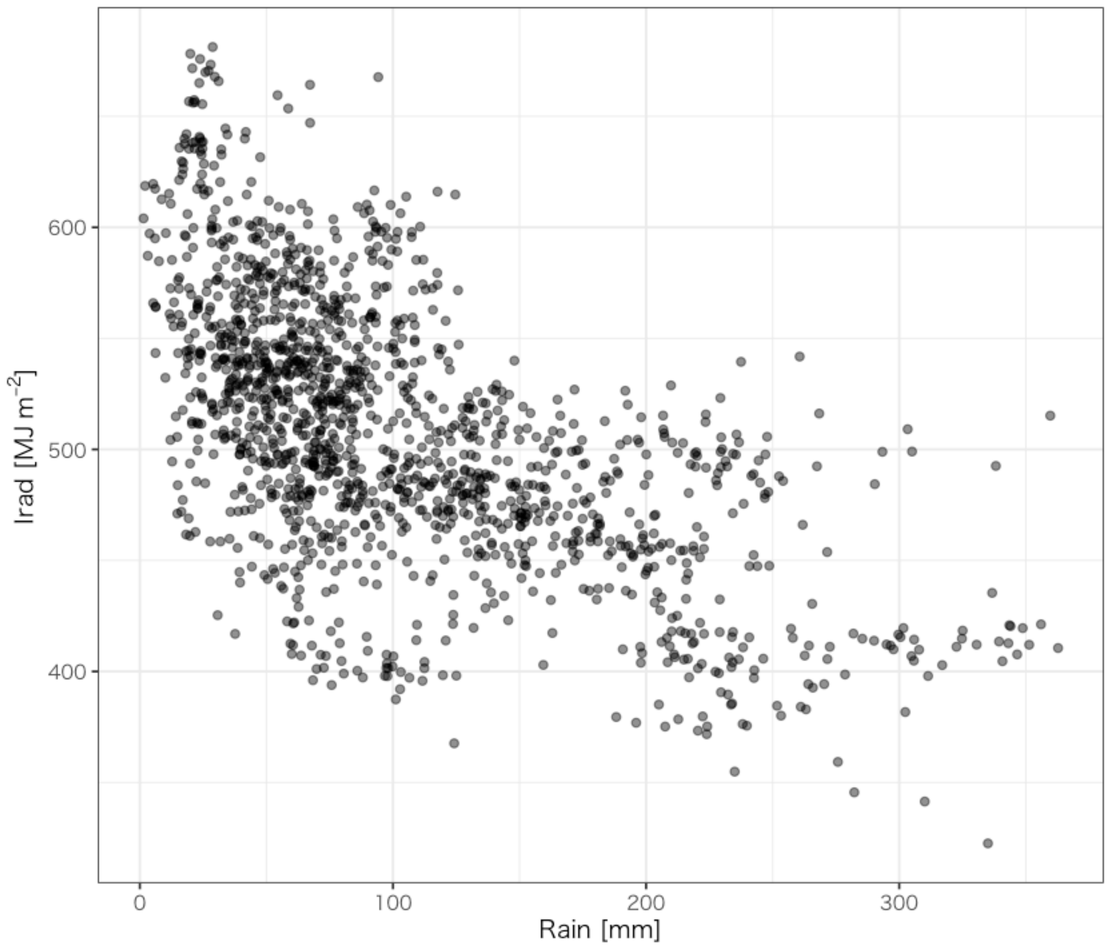

Figure S4: Relationship between total precipitation and solar irradiance during the grain-filling period (late June to mid July).

 doi: 10.1371/journal.pone.0258677

Table S1: List of hyperparameters tuned to improve model performance.

Base learner	Parameter 1	Parameter 2
Partial least squares	[1]ncomp = {1, 2, 3}	
Support vector machine	[2]C = {0.25, 0.50, 1.00}	[3]sigma = {-}
Random forest	[4]mtry = {2, 20, 39}	
Cubist	[5]comittees = {1, 10, 20}	[6]neighbors = {0, 5, 9}
Neural network	[7]size = {1, 3, 5}	[8]decay = $\{0, 10^{-1}, 10^{-4}\}$

[1] number of components

[2] cost of constraints violation

[3] kernel parameter automatically calculated from the training data

[4] number of variables randomly sampled as candidates at each split

[5] number of members of the ensemble

[6] number of training set instances that are used to adjust the model-based prediction.

[7] number of units in the hidden layer

[8] parameter for weight decay
